# Finding a sensible approach to sensitive data

**DOI:** 10.1038/sdata.2018.253

**Published:** 2018-11-06

Today the journal released a checklist for researchers submitting descriptions of data derived from human subjects. The checklist is designed to make it easier for authors to understand and comply with our policies, while also making any restrictions on the data more transparent to the journal’s editors and staff.

Datasets derived from human patients or participants must often be distributed under special conditions in order to enforce ethical and legal use of the data, and to protect privacy. Peer review of these datasets can be inherently challenging.

When authors submit descriptions of sensitive human datasets, we initiate a conversation with them and their relevant ethics officers to identify a safe and secure way for peer review to occur. The checklist we have released today is designed to help facilitate this conversation. Authors who are considering submitting a description of a human-derived dataset should review this checklist and contact us early to begin this conversation. The checklist is now available online (https://go.nature.com/2Nn9Kkx).

Key parts of this checklist have been developed based on an editorial policy checklist already in use at the Nature-titled journals^1^. Our checklist additionally asks authors to provide detailed information on how data will be made accessible during peer-review and after publication.

Since launch, the journal has been working to develop sensible policies that allow us to evaluate and publish sensitive human-derived datasets in a thorough manner^2,3^. First, we ask that the data be hosted in a repository that has experience handling and sharing sensitive data. Our repository list now includes a series of recommendations (https://go.nature.com/2IE24tl). Second, there must be a viable and well-described mechanism by which other qualified researchers can apply for access to the data. Conditions should permit competitive reuse and should not limit reuse only to collaborators of the authors. Lastly, our referees should be able to access the data in a timely manner and without revealing their identities. This last point is often the most difficult to arrange.

If there is no way for our referees to safely access a dataset, we will generally decline the submission. Data review, after all, is a central pillar of the journal’s editorial process.
